# The prevalence of psychological distress and its association with coping strategies among medical interns in Malaysia: A national‐level cross‐sectional study

**DOI:** 10.1111/appy.12417

**Published:** 2020-08-26

**Authors:** Munirah Ismail, Kun Yun Lee, Afandy Sutrisno Tanjung, Ida Anum Ahmad Jelani, Rabiah Abdul Latiff, Hashimah Abdul Razak, Nor Izzah Ahmad Shauki

**Affiliations:** ^1^ Institute for Health Management Ministry of Health Shah Alam Malaysia; ^2^ Wilayah Persekutuan Kuala Lumpur & Putrajaya State Health Department Ministry of Health Kuala Lumpur Malaysia; ^3^ Kedah State Health Department Ministry of Health Alor Setar Malaysia; ^4^ Hospital Tawau Ministry of Health Tawau Malaysia; ^5^ Hospital Kuala Lumpur Ministry of Health Kuala Lumpur Malaysia; ^6^ Institute for Health Systems Research Ministry of Health Shah Alam Malaysia

**Keywords:** anxiety, COPE, depression, housemanship, stress

## Abstract

**Introduction:**

The prevalence of psychological distress is increasing worldwide. Stressful working environments and high expectations in medical practice put doctors at high risk of depression, anxiety, and stress, especially among medical interns. Effective coping strategies may reduce psychological distress in the clinical setting. This study aimed to determine the prevalence of psychological distress and its association with coping strategies among medical interns in Malaysia.

**Method:**

A total of 431 medical interns at 26 Malaysian Ministry of Health hospitals participated in this cross‐sectional study in 2017. Self‐administered questionnaires consisting of sociodemographic characteristics, items from DASS and BRIEF COPE were used. Descriptive analysis was done followed by further analysis with chi‐square and Spearman correlation tests.

**Results:**

The prevalence of stress, anxiety, and depression was 29.7%, 39.9%, and 26.2%, with a significantly higher prevalence among female and younger interns. Three‐quarters of them (73.1%) applied problem‐focused strategies as the main coping mechanism. Emotion‐focused coping strategies showed a significant but weak correlation with anxiety and stress whereas avoidance‐based coping strategies were significantly correlated with depression, anxiety, and stress.

**Discussion:**

Medical internship is a highly grueling period. Besides emphasizing clinical competency, internship training should also impart the practice of healthy coping mechanisms. The vulnerable groups of females and younger interns should be taught positive coping skills so that they are empowered to handle any stressors on their personal and professional lives. Optimum psychological wellbeing of the medical interns can improve the overall work performance and quality of care for patients.

## 1 INTRODUCTION

Psychological distress such as depression, anxiety, and stress are increasingly prevalent nowadays. Worldwide, more than 450 million people were found to have mental problems. The disease burden related to mental health is expected to increase to 15% by 2020 (World Health Organisation, 2001). In recent years, mental health issues are increasingly recognized as a major public health problem that affect physical wellbeing and result in a wide range of social and economic implications (World Health Organization, 2004). The Mental Health Action Plan 2013‐2020 advocated four main objectives to reduce mental illness‐related mortality and morbidity, namely effective leadership and governance for mental health, the provision of comprehensive mental health and social care services in community‐based settings, implementation of strategies for promotion and prevention, and strengthening information systems, evidence, and research (World Health Organization, 2013). In Malaysia, according to the National Health and Morbidity Survey 2015, one in three (29.2%) adults are affected by mental health problems, a prevalence rate that increased from 10.7% in 1996 (Institute for Public Health, 2015).

Multiple factors contribute to the development of mental illness, ranging from biological, psychological, socioeconomic, and lifestyle factors (Bergdahl & Bergdahl, 2002; Carod‐Artal, 2017; Walsh, 2011). Occupation‐related factors such as high job demand, low job control, and poor work support have been linked with adverse physical and mental health effects issues such as anxiety, depression, and fatigue (Cheng, Guo, & Yeh, 2001; Karasek, 1979; Lorant et al., 2003; Sargent & Terry, 2000). In medical practice, healthcare professionals (HCP) work in a highly stressful environment involving high patient load and long working hours, predisposing many of them to stress, anxiety, and depression. In severe cases, some might even resort to substance abuse and addiction (Cifuentes et al., 2008; Sundquist & Johansson, 2000). HCPs report a higher level of psychological distress (28%) compared to the general population (14%‐18%) (Firth‐Cozens, 2001; Kroenke, Spitzer, Williams, Monahan, & Lowe, 2007; Wall, Bolshaw, & Carolan, 2006). Doctors with poor mental health may progress to developing psychiatric disorders which further compromise their quality of life, work performance, and quality of care for patients.

In Malaysia, new medical graduates need to undergo a compulsory 2‐year internship in government hospitals to gain sufficient knowledge and skills that meet the standard of the medical profession (Malaysian Medical Council, 2008). The medical internship involves 2 years of training rotation in six clinical departments, including 4 months each in Medicine, Surgery, Orthopedic, Pediatrics, Obstetrics & Gynecology, with the sixth posting in either Accident & Emergency/Anesthesiology/Psychiatry/Primary Care. Yusoff et al. (2013) reported that one in three (31%) interns in a Malaysian university hospital experienced stress due to fear of committing mistakes, high workload, and uncooperative colleagues. Another study in an East Malaysian training hospital reported stress, anxiety, and depression to be common among interns, with prevalence rates of 57%, 63.7%, and 12.9% respectively (Shahruddin et al., 2016). In Malaysia, doctors with psychiatric illnesses will be reviewed by the Medical Review Panel of the Malaysian Medical Council on their fitness to practice to ensure patient safety. Among the cases reviewed in 2015, the diagnosis encountered were depressive disorders (40%), adjustment disorders (22%), and anxiety disorder (9%). Half of the cases (53%) referred to MRP were interns in their first posting (Ismail, Zakaria, Majimbun, Bidin, & Ahmad Shauki, 2016).

This trend is particularly worrying because psychologically distressed doctors may not be able to deliver the best patient care. Junior doctors at the beginning of their careers are most at risk as they have to cope with long working hours and lack of sleep. They also struggle to adapt to challenging and emotional situations in hospitals (Sen et al., 2010). However, some researchers have proposed that the underlying coping strategies could influence how different individuals deal with stressful environments. Lazarus and Folkman (1987) described three coping styles. Firstly, problem‐focused coping uses action‐focused mechanism to manage the issues surrounding the stressors. Persons with this coping style would attempt to alter the stressor, for example, learning new skills to solve a problem, planning and making appropriate decisions to resolve conflicts. Secondly, individuals with emotion‐focused coping strategies would lessen negative emotions during a distressful situation by accepting the inevitable or seeking support from peers or family members. Lastly, persons with avoidance coping skills focus on ignoring the stressors, making them passive and negative in dealing with the stressors (Holahan, Holahan, Moos, Brennan, & Schutte, 2005). Active problem solving during stressful situations has been shown to improve academic performance and better health (Struthers, Perry, & Menec, 2000). The effective use of coping strategies may offset the negative impacts of internship and facilitate the learning and adaptation process.

In view of this, it is vital to ascertain the role of coping strategies in overcoming possible stressors that may lead to psychological distress among medical interns. To date, most of the studies from Malaysia focused on a single psychological distress condition or were based in a single center. There is a lack of national‐level data on the prevalence of psychological distress. Therefore, this study aims to determine the prevalence of psychological distress and its association with coping strategies among medical interns in Malaysia. The outcomes from this study would serve as important guidance to policymakers to undertake strategies in improving the working conditions and internship training system.

## 2 METHOD

This multi‐center cross‐sectional study was conducted from June to October 2017 in 26 Malaysian government hospitals that provided internship training during the same period. To qualify as a medical officer, graduated medical students need to complete six postings of 4‐month duration in different clinical departments (Malaysian Medical Council, 2008). In this study, all interns who were the first and second year of training were included in the sampling frame. However, those on psychiatric treatment were excluded.

The sample size was calculated based on a published local study that reported the prevalence of stress, anxiety, and depression among interns to be 56%, 57%, and 44% (Hussain & Nordin, 2012). To achieve the sample size, absolute precision of 5% of the true proportion at 95% confidence was used with the prevalence of depression of 44%. With the expected response rate set at 70%, the required sample size estimated to be 379. It was further inflated by 10% to 417 to accommodate any missing data.

$$n=\left[z^{2}\times p\times \left(1-p\right)\right]/d^{2}=\left[1.962\times \left(0.44\right)\times \left(0.56\right)\right]/0.052=378.63=379z=1.96\left(95\%\text{Confidence Interval}\right),d=\text{degree of accuracy=0.05}p=\text{Prevalence of self}-\text{perceived depression}=44\%\text{Minimum sample size required},N=379\times 110\%=416.9=417.$$

The complete name list of interns was obtained from the Human Resource Manager of the respective hospitals. Systematic random sampling was employed to recruit 20 interns from each hospital. An equal proportion of interns from Year 1 and Year 2 of training were invited to the data collection session. A short briefing about the research objective was given before consent forms were distributed. All respondents were informed that their responses would remain confidential. Following that, the respondents took turns to answer the questionnaires that had been pre‐installed in computer tablets.

The questionnaire consisted of three sections. Section 1 included baseline sociodemographic characteristics such as age, gender, ethnicity, religion, marital status, and year of internship. Section 2 adapted the original English version DASS‐42 questionnaire (Lovibond & Lovibond, 1995). It consists of 42 items categorized into 3 sub‐scales, namely depression, anxiety, and stress. The respondent is required to indicate the presence of a symptom during the past 7 days. Each item in the questionnaire is formulated for responses based on a 4‐point Likert scales; ranging from 0‐Did not apply to me at all to 3‐Applied to me very much or most of the time. The sum of the scores for each question in every sub‐scale is computed and evaluated as per the severity categories of normal, mild, moderate, severe, and extremely severe. Higher scores indicated a higher level of severity in each dimension. The validity and reliability of DASS‐42 have been assessed among healthcare and medical populations in previous research (Nieuwenhuijsen, De Boer, Verbeek, Blonk, & Van Dijk, 2003).

In Section 3, Brief COPE (Carver, 1997), a shortened version of the COPE Inventory (Carver, Scheier, & Weintraub, 1989), was used to measure the coping strategies of the respondents. It is a multidimensional scale with 2 items under each of the 14 subscales. It assesses different coping strategies, including self‐distraction, active coping, denial, use of instrumental support, substance use, use of emotional support, positive reframing, behavioral disengagement, venting, planning, humor, acceptance, religion, and self‐blame. The response for each item is measured on a Likert scale from 1‐“I haven't been doing this at all,” 2‐“I've been doing this a little bit,” 3‐“I've been doing this a medium amount,” to 4‐“I've been doing this a lot”. Brief COPE has been translated into various languages and used in different countries. Its internal reliability and factor structure have been shown to be in line with the full COPE (Baumstarck et al., 2017; Monzani et al., 2015; Tang, Chan, Ng, & Yip, 2016).

All the data collected were cleaned and analyzed using SPSS version 22. Descriptive analysis was performed to describe the sociodemographic characteristics of the respondents besides their coping strategies and psychological distress. Chi‐square test was used to examine the association between the sociodemographic variables and the psychological distress while Spearman correlation test was conducted to delineate the association between coping strategies and psychological distress. The level of statistical significance was set at *P* < .05 for all analyses. The study was registered with the National Medical Research Registry (NMRR‐16‐1230‐29844) and received a Ministry of Health (MOH) research grant. Ethical approval was obtained from the Malaysia Medical Research Ethics Council.

## 3 RESULTS

From the 520 interns recruited at the 26 hospitals, 431 completed questionnaires were obtained and included in the final analysis. The remaining 89 respondents were excluded because of refusal to participate and incomplete data. Table 1 outlines the sociodemographic characteristics of the 431 respondents. Every three out of the four interns (74.0%) were between 26‐30 years old and more than half (60.3%) were females. In terms of ethnicity and religions, the distribution of the respondents closely mirrored the underlying population distribution in the country. Only one‐quarter of the interns (24.6%) in the study were married. Approximately half of the respondents were in the first (47.3%) and second (52.7%) years of training respectively.

**TABLE 1:** Baseline socio‐demographic characteristics of respondents

Variables	n	%
**Age Group**		
20‐25	104	24.1
26‐30	319	74.0
31‐35	8	1.9
**Gender**		
Male	171	39.7
Female	260	60.3
**Ethnicity**		
Malay	285	66.1
Chinese	89	20.6
Indian	45	10.4
Others	12	2.8
**Religion**		
Islam	290	67.3
Buddha	62	14.4
Hindu	39	9.0
Christian	34	7.9
Others	6	1.4
**Marital Status**		
Single	325	75.4
Married	106	24.6
**Year of Internship Training**		
First Year	204	47.3
Second Year	227	52.7

Table 2 shows the distribution and ranking of coping strategies based on Brief‐COPE. Three‐quarters of the surveyed interns (73.1%) applied problem‐focused strategies as the main coping mechanism. Only a small number of the respondents resorted to emotion‐focused (14.4%) and avoidance (4.4%) strategies. After removing the 35 respondents with a tied score, the mean score of the 14 items in Brief COPE was calculated. Based on the ranking, the most commonly adopted coping strategies were acceptance, followed by positive reframing and religion. Apart from self‐distraction, most of the respondents did not resort to avoidance coping strategies. The lowest mean scores were recorded for behavioral disengagement, denial, and substance abuse.

**TABLE 2:** Distribution and ranking of coping strategies based on brief‐COPE among the respondents

Coping strategy	n	%
**Category**		
Problem‐focused	315	73.1
Emotion‐focused	62	14.4
Avoidance	19	4.4
Tied Score	35	8.1

Score by category	Mean	SD
**Emotion‐focused**	27.3	5.12
Acceptance	6.5	1.44
Positive Reframing	6.2	1.54
Religion	6.2	1.86
Emotional Support	5.6	1.72
Humor	4.5	1.79
**Problem‐focused**	23.8	4.84
Active Coping	6.1	1.47
Planning	5.9	1.49
Instrumental Support	5.7	1.80
**Avoidance**	18.6	4.10
Self‐Distraction	6.0	1.48
Venting	4.8	1.58
Self‐Blame	4.5	1.65
Behavioral Disengagement	3.1	1.43
Denial	2.9	1.28
Substance Use	2.1	0.64

Table 3 shows the psychological distress status of the respondents in terms of depression, anxiety, and stress. Overall, the majority of the respondents reported a normal level of mental wellbeing. The prevalence of stress, anxiety, and depression was 29.7%, 39.9%, and 26.2%. Most of them were at the mild and moderate levels of depression, anxiety, and depression. Only a small proportion was at the severe and very severe levels. Among the three types of psychological distress, the mean score of stress was the highest (11.6 ± 8.42), followed by anxiety (7.9 ± 7.05), and depression (7.4 ± 8.53).

**TABLE 3:** Distribution of psychological distress of depression, anxiety, and stress based on DASS‐42 among the respondents (n = 431)

	Depression		Anxiety		Stress	
Mean ± SD	7.4 ± 8.53		7.9 ± 7.05		11.6 ± 8.42	
**Level**	**n**	**%**	**n**	**%**	**n**	**%**
Normal	318	73.8	259	60.1	303	70.3
^a^Mild	43	10.0	46	10.7	52	12.1
^a^Moderate	32	7.4	69	16.0	42	9.7
^a^Severe	17	3.9	18	4.2	25	5.8
^a^Very Severe	21	4.9	39	9.0	9	2.1

^a^ Score > normal level considered as screened positive for depression/anxiety/stress under DASS.

Table 4 shows the results of the Chi‐square test on the association between the sociodemographic factors and psychological distress. Depression, anxiety, and stress were significantly higher among female respondents (*P* < .05). The prevalence of anxiety and stress were also significantly higher among respondents from the younger age group (age 25‐30). None of the respondents in the age group of 31‐35 years were screened positive for depression, anxiety, or stress. No other significant associations were detected among the other variables.

**TABLE 4:** Association of psychological distress based on DASS‐42 with sociodemographic characteristics among the respondents

	Depression			Anxiety			Stress		
	n	%	*P* value	n	%	*P* value	n	%	*P* value
**Age Group**			**.096**			**.028**			**.019**
20‐25	33	31.7		48	46.2		40	38.5	
26‐30	80	25.1		124	38.9		88	27.6	
31‐35	0	0.0		0	0.0		0	0.0	
**Gender**			**.048**			**.000**			**.011**
Male	36	21.1		49	28.7		39	22.8	
Female	77	29.6		123	47.3		89	34.2	
**Ethnicity**			**.245**			**.124**			**.109**
Malay	75	26.3		118	41.4		87	30.5	
Chinese	20	22.5		27	30.3		22	24.7	
Indian	12	26.7		20	44.4		12	26.7	
Others	6	50.0		7	58.3		7	58.3	
**Religion**			**.265**			**.063**			**.593**
Islam	79	27.2		122	42.1		90	31.0	
Buddhism	10	16.1		18	29.0		14	22.6	
Hinduism	11	28.2		19	48.7		11	28.2	
Christian	12	35.3		13	38.2		12	35.3	
Others	1	16.7		0	0.0		1	16.7	
**Marital Status**			**.958**			**.451**		**0.906**	
Single/Divorced	85	26.2		133	40.9		97	29.8	
Married	28	26.4		39	36.8		31	29.2	
**Year of Internship Training**			**.441**			**.194**			**.176**
Year 1	57	27.9		88	43.1		67	32.8	
Year 2	56	24.7		84	37.0		61	26.9	

The association between the scores of coping strategies and psychological distress was determined using the Spearman correlation test due to the non‐parametric distribution (Table 5). r value <0.20 was considered as weak correlation, 0.2 < r < 0.8 was considered as moderate correlation, and r > 0.8 was considered as strong correlation. Respondents who applied emotion‐focused coping strategies had a significant but weak correlation with anxiety and stress (*P* < .001). However, the interns who resorted to avoidance‐based coping strategies reported a significant moderate correlation with depression, anxiety, and stress (Table 5).

**TABLE 5:** Correlation between preferred coping strategies and psychological distress

	Depression		Anxiety		Stress	
Coping strategy	r	*P* value	r	*P* value	r	*P* value
Problem‐focused	−0.040	.410	0.080	.098	0.042	.385
Emotion‐focused	0.084	.080	0.185	**.000**	0.195	**.000**
Avoidance	0.570	**.000**	0.530	**.000**	0.563	**.000**

## 4 DISCUSSION

This study examined the prevalence of stress, anxiety, and depression and its association with coping strategies among the medical interns in Malaysia. The findings showed that 39.9% of interns experienced anxiety, followed by stress and depression at 29.7% and 26.2%. This finding showed a slight difference as compared to previous studies in which 63% and 31% of interns experienced anxiety and stress (Shahruddin et al., 2016; Yusoff, Tan, & Esa, 2011). Aside from methodological differences, the lower prevalence of anxiety and stress in this study might be attributed to the various improvement strategies in the internship training over the last few years, such as the mentor‐mentee program and modified flexi‐shift system. By comparison, the prevalence of anxiety among junior doctors in an Australian study was higher (42.1% in 2014) than this study (Soares & Chan, 2016). Similar findings were reported in Pakistan (43.7%), India (56%), and Saudi Arabia (73%) (Abdulghani et al., 2014; Rab, Mamdou, & Nasir, 2008; Singh & Jha, 2013). In other words, mental health problems among junior doctors is a prevailing issue across developed and developing countries.

Furthermore, this study also found that females and younger interns were significantly more predisposed to psychological distress. First‐year interns reported a higher level of stress, anxiety, and depression compared to their seniors in the second year of internship. A similar study in a local university hospital revealed no associations between anxiety and sociodemographic characteristics including gender, ethnicity, marital status, and seniority (Tan et al., 2013). Other published studies on the relationship between psychological wellbeing and gender differences among medical students showed mixed findings (Bayram & Bilgel, 2008; Saravanan & Wilks, 2014; Zaid, Chan, & Ho, 2007). Nevertheless, studies on practicing doctors in other countries reported similar findings in which female doctors were more likely to report psychological distress, especially anxiety symptoms (Erdur et al., 2006; Thomas, 2004; Wang et al., 2011). One possible reason is the innate nature of females being more competitive and concern about their performance, thus putting them at a higher risk of developing negative mental states (Inam, Saqib, & Alam, 2003).

The age of interns and year of training they are in signify their maturity and seniority in the healthcare system. Newcomers in the system need to adjust to a new environment and to handle academic and professional expectations of their training (Uehara, Takeuchi, Kubota, Oshima, & Ishikawa, 2010). This is especially true for newly graduated medical students entering internship training. They need to apply the theory and knowledge learned in medical schools into the clinical practice in the hospital setting. Senior doctors' high expectations on them can be a stressor to many interns. If poorly handled, it may culminate in psychological distress. While continuous support should be in place at the workplace for all levels of HCP, targeted intervention in the form of reinforcement of healthy coping mechanisms is especially needed for the interns during the initial grueling period.

Coping researchers have provided evidence of the role of coping strategies in work‐related stress (Shapiro, Astin, Bishop, & Cordova, 2005; Wallace, Lemaire, & Ghali, 2009). For example, the levels of depression, anxiety, and stress among students can be predicted by the choice and use of coping strategies when encountering problems (Mahmoud, Staten, Hall, & Lennie, 2012). In this study, most of the interns used the problem‐focused coping strategy and only a small number resorted to avoidance coping style. Interns who applied avoidance‐based coping strategies showed a moderate association with depression, anxiety, and stress. By actively trying to find solutions to reduce stress, the persons are less prone to burnout as compared to those who did nothing about their stress (Maslach, Schaufeli, & Leiter, 2001). Rodham and Bell (2002) found that nurses who used problem‐oriented coping were able to handle stress well and they had greater job satisfaction. A study conducted among primary care doctors found useful strategies for doctors to deal with stress at work such as talking with colleagues, humor, physical activity, family interaction, and spending time alone (Lemaire & Wallace, 2010). It is very important to educate junior doctors on the effective use of positive coping behaviors to prevent the development of psychological distress such as depression, anxiety, and stress.

On a further note, stressful working environment and long working hours are closely associated with physical illness, poor mental health, substance abuse, and poor performance. As a result, there is a higher tendency in clinical errors, leading to suboptimal care for patients (Fahrenkopf et al., 2008; Perera, Torabi, & Kay, 2011; Rada & Johnson‐Leong, 2004; Vidyarthi, Auerbach, Wachter, & Katz, 2007; Wirtz, Nachreiner, & Rolfes, 2011). One of the most worrying phenomena is substance abuse among HCP. The rate of substance abuse reported in this study was lower than similar studies conducted in South Africa and Mexico, whereby one‐fifth of respondents admitted to the consumption of alcohol or other illicit substances to cope with their medical internship. Despite the self‐reported low rate of substance abuse in this study, other negative coping mechanisms may lead to unresolved issues that predispose the individuals are prone to develop serious mental health disorders (Gao et al., 2012). In view of that, apart from identifying and alleviating the stressors, it is also important to provide holistic training during the internship by emphasizing on resilience building and positive coping besides focusing merely on clinical competency. Efforts to educate interns on applying healthy coping mechanisms in stressful situations were highlighted in the “Guidebook for House Officers” (Malaysian Medical Council, 2008). However, more comprehensive and effective measures must be put in place to empower the interns with the appropriate coping skills to reduce the disruptive effects of any stressors on their personal and professional lives.

There are several limitations to this study. The cross‐sectional study design identified only associations and thus no causal inferences can be made among the studied outcome variables. Furthermore, the application of self‐administered questionnaires may lead to reporting bias and social desirability bias, resulting in the under‐ or over‐reporting of their actual conditions. Despite these limitations, this is the first national‐level study in Malaysia to measure the prevalence of stress, anxiety, and depression and their associated factors among interns in Malaysia. By including the evaluation of coping strategies applied by the interns, the findings are beneficial for policymakers to implement the necessary improvement on the internship training system.

## 5 CONCLUSION

The medical internship is a challenging period for any young doctor. While it may not be entirely possible to prevent stress, depression, and anxiety, it is vital for the relevant stakeholder to acknowledge the rising prevalence of psychological distress among interns so that necessary strategies can be implemented to safeguard their personal psychological wellbeing and quality patient outcomes. The reinforcement of healthy coping strategies should be a priority in improving the mental wellbeing of HCP, especially junior doctors. Short and long‐term support strategies made available at various levels of the healthcare system would be beneficial to reduce any psychological distress. Further research may benefit from longitudinal study designs with a more comprehensive set of demographics, personal, and work‐related factors to fully determine the predictors of negative mental states in different groups of HCPs.

## CONFLICT OF INTEREST

No potential conflict of interest was reported by the authors.

## AUTHOR CONTRIBUTIONS

M.I., A.S.T., I.A.A.J., R.A.L., H.A.R., and N.I.A.S. conceived the overall study. All authors are involved in the design of the study. A.S.T., I.A.A.J., R.A.L., and H.A.R. coordinated the data collection. All authors are involved in the data analysis. M.I. and K.Y.L. drafted the manuscript. All authors read and approved the final manuscript.

## Data Availability

The data that support the findings of this study are available from the corresponding author upon reasonable request.
